# Impact of BMI, Physical Activity, and Sitting Time Levels on Health-Related Outcomes in a Group of Overweight and Obese Adults with and without Type 2 Diabetes

**DOI:** 10.3390/jfmk7010012

**Published:** 2022-01-17

**Authors:** Roberto Pippi, Lucia Cugusi, Marco Bergamin, Vittorio Bini, Carmine Giuseppe Fanelli, Valentina Bullo, Stefano Gobbo, Andrea Di Blasio

**Affiliations:** 1 Healthy Lifestyle Institute, C.U.R.I.A.Mo. (Centro Universitario Ricerca Interdipartimentale Attività Motoria), Department of Medicine and Surgery, University of Perugia, Via G. Bambagioni, 19, 06126 Perugia, Italy; carmine.fanelli@unipg.it; 2 Department of Biomedical Sciences, University of Sassari, 07100 Sassari, Italy; lcugusi@uniss.it; 3 Department of Medicine, Sport and Exercise Medicine Division, University of Padova, Via Giustiniani 2, 35128 Padova, Italy; marco.bergamin@unipd.it (M.B.); valentina.bullo@unipd.it (V.B.); stefano.gobbo@unipd.it (S.G.); 4 Department of Medicine and Surgery, University of Perugia, Via Gambuli, 1, 06132 Perugia, Italy; vittorio.bini@unipg.it; 5 Department of Medicine and Aging Sciences, ‘G. D’Annunzio’ University of Chieti-Pescara, 66100 Chieti, Italy; andrea.diblasio@unich.it

**Keywords:** physical activity level, sitting time, body mass index, exercise, obesity, type 2 diabetes

## Abstract

Physical activity level and sedentary behaviors affect health status in people with obesity and type 2 diabetes (DM2); their assessment is mandatory to properly prescribe exercise programs. From January 2011 to February 2014, 293 overweight/obese adults (165 women and 128 men, mean age of 51.9 ± 9.5 years and 54.6 ± 8.3 years, respectively), with and without DM2, participated in a three-month intensive exercise program. Before starting, participants were allocated into three subgroups (overweight, body mass index or BMI = 25–29.9; class 1 of obesity, BMI = 30–34.4; or class 2 (or superior) of obesity, BMI > 35). The international physical activity questionnaire (IPAQ-it) was used to evaluate participants’ baseline sitting time (SIT) and physical activity level (PAL). Stratified multiple analyses were performed using four subgroups of SIT level according to Ekelund et al., 2016 (low, 8 h/day of SIT) and three subgroups for PAL (high, moderate, and low). Health-related measures such as anthropometric variables, body composition, hematic parameters, blood pressure values, and functional capacities were studied at the beginning and at the end of the training period. An overall improvement of PAL was observed in the entire sample following the three-month intensive exercise program together with a general improvement in several health-related measures. The BMI group factor influenced the VO_2_ max variations, leg press values, triglycerides, and anthropometric variables, while the SIT group factor impacted the sitting time, VO_2_ max, glycemic profile, and fat mass. In this study, baseline PAL and SIT did not seem to influence the effects of an exercise intervention. The characteristics of our educational program, which also included a physical exercise protocol, allowed us to obtain positive results.

## 1. Introduction

It is well established that physical activity level (PAL) and sedentary behaviors affect people’s health status [1], especially in populations with obesity and type 2 diabetes (DM2) [2]. The World Health Organization (WHO) has stated that “adults should do at least 150–300 min of moderate-intensity aerobic physical activity; or at least 75–150 min of vigorous-intensity aerobic physical activity; or an equivalent combination of moderate- and vigorous-intensity physical activity (MVPA) throughout the week, for substantial health benefits”. Additionally, muscle-strengthening activities, involving all major muscle groups, on two or more days per week at moderate or greater intensity are recommended. People who do not engage in at least 150 min per week of MVPA are defined as inactive [3]. Additionally, sedentariness, defined as “any waking behavior characterized by an energy expenditure ≤1.5 METs, while in a sitting, reclining, or lying posture” [4,5,6], including sitting time (SIT), is a relevant health problem [7] as it is associated with an increase of cardio-metabolic risk [8], obesity, and DM2 [9]. Moreover, literature reports the importance of both SIT and PAL for promoting metabolic health [8,10,11]. In this regard, the WHO recently provided evidence-based public health recommendations for people of all ages on the amount of physical activity, sedentary behavior, and health outcomes [4]. To apply the WHO guidelines, it is crucial to promote interventions aimed at increased levels of physical activity and to monitor trends in both physical activity and SIT, including by low-cost and reliable measuring tools of habitual physical activity, such as questionnaires [12]. In this regard, the International Physical Activity Questionnaire in Short Form (IPAQ-SF) is a validated and most widely used [13] physical activity questionnaire [14].

Previous studies have emphasized the importance of physical exercise type for the optimization of results, recommending prescribing physical exercise for the management of obesity-related comorbidities [15,16,17] and DM2 [18,19,20,21,22,23]. Moreover, a part of the literature reports the presence of a negative compensation for spontaneous physical activity, with the inclusion of physical exercise in inactive people, based on the baseline BMI and PAL [24]. The purpose of this study was to evaluate the effects of an intensive exercise program on health-related outcomes (e.g., body mass index or BMI, waist circumference or WC, body composition, muscular strength, and maximal oxygen consumption or VO_2_ max) and cardio-metabolic health measures (e.g., blood pressure levels, lipids, and glycemic profile) in a group of overweight and obese adults with and without DM2. We also studied whether, at the end of the exercise period, changes differed among different BMI, PAL, and SIT baseline categories.

## 2. Materials and Methods

### 2.1. Participants

**Figure 1:**
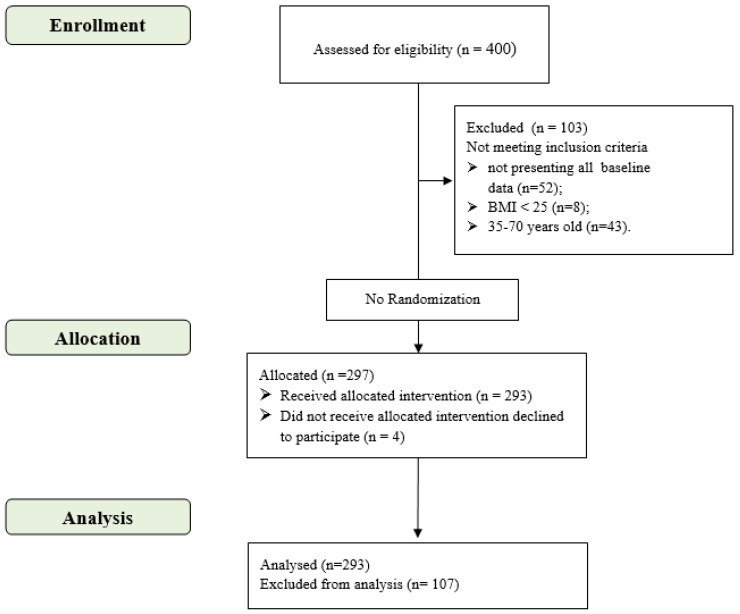
CONSORT flow diagram (adapted from CONSORT 2010 flow diagram).

From January 2011 to February 2014, a total sample of 293 (Figure 1) overweight/obese adults (165 women and 128 men, mean age 51.9 ± 9.5 years and 54.6 ± 8.3 years, respectively) with and without DM2 were recruited at the C.U.R.I.A.Mo. center to follow an intensive and multidisciplinary intervention protocol, as described by De Feo et al. [25].

Inclusion criteria were the presence of all data from clinical, anthropometric, and self-report questionnaires and physical measures collected both before and after the intervention; age between 35 and 70 years, and a BMI ≥ 25 kg/m^2^. The exclusion criteria were the presence of musculoskeletal disorders or other clinical conditions that could seriously reduce subjects’ life expectancy or their ability to participate in the study, particularly any potential contraindications to exercise.

According to the WHO criteria [26], participants were allocated into three subgroups based on baseline BMI value (Table S1a). The resultant groups were as follows:

- “people with overweight” (or OVER), corresponding to BMI 25–29.9 (n = 63, BMI mean = 28 ± 1.31);
- “people with I degree of obesity” (or I OB), corresponding to BMI 30–34.9 (n = 131, BMI mean = 32.5 ± 1.48);
- “people with II degrees (or superior) of obesity” (or II OB), corresponding to BMI > 35 (n = 99, BMI mean = 38.6 ± 3).

According to IPAQ guidelines [27], participants were further allocated into three resultant subgroups with reference to the baseline level of PAL (Table S1b), as follows:

- “low PAL”, (n = 153, mean = 2.2 ± 2.8 MET-h per week);
- “moderate PAL”, (n = 108, mean = 20.4 ± 11.8 MET-h per week);
- “high PAL”, (n = 32, mean = 71.4 ± 32.7 MET-h per week).

Finally, according to Ekelund et al. (2016) [10], participants were allocated into four categories on baseline levels of SIT (Table S1c). This resulted in the following groups:

- “low SIT”, corresponding to <4 h/day (n = 82, mean = 1.5 ± 1.2 h/day);
- “medium SIT”, corresponding to 4–5.9 h/day (n = 63, mean = 4.6 ± 0.5 h/day);
- “high SIT”, corresponding to 6–8 h/day of sitting time (n = 99, mean = 6.8 ± 0.8 h/day);
- “very high SIT”, corresponding to >8 h/day of sitting time (n = 43, mean = 10.4 ± 1.5 h/day).

Please see Table S1a–c for the baseline mean values of all parameters in the entire sample and in the subgroups.

### 2.2. Intervention

Participants were involved in a three-month physical activity habits intervention, including one individual medical examination conducted by an endocrinologist; one psychological interview focused on lifestyle changes with a psychologist; one individual nutritional, counseling session focused on nutritional habits; and an intensive, gym-based, exercise intervention program. Briefly, as reported by Pippi et al. (2020) [28], the exercise program consisted of 25 bi-weekly small-group practical and counseling sessions (five persons per group), conducted by a certified exercise specialist. Every session lasted 90 min and including a mix of aerobic and strength exercise, administered using the circuit training method.

This approach derived from the original C.U.R.I.A.Mo. clinical model protocol, previously described by De Feo et al. [25]. Briefly, this clinical model utilizes the participation of master trained specialists who work following a multidisciplinary method. It aims to decrease sedentary time by guiding the patient to gradually increase intentional physical activity. The C.U.R.I.A.Mo. project has been registered in the Australian New Zealand Clinical Trials Registry (a Primary Registry in the WHO registry network), with the number: ACTRN12611000255987.

All the participants gave their written informed consent to participate in the study. Using a quasi-experimental study design, individuals were assessed before (T0) and at the end of the multidisciplinary intervention (T1).

### 2.3. Measures

#### 2.3.1. Clinical and Anthropometric Variable Measures

During the first medical examination, managed by the endocrinologist, clinical variables including systolic (SBP) and diastolic (DBP) blood pressure (through a UM-101 mercury-free sphygmomanometer, A&D Medical, Tokyo, Japan) as well as blood measures such as fasting plasma glucose (GLYC), hemoglobin A1c (HbA1c), total (COL), high-density (HDL) and low-density lipoprotein (LDL) cholesterol, and triglycerides (TRIG), were recorded according to national standards of care [29]. Moreover, anthropometric measures (e.g., weight, BMI, WC) and body composition (fat mass percentage or FM% and muscle mass or MM) were assessed using standard methods with the Tanita body composition analyzer BC-420MA (Tokyo, Japan). Finally, during the medical examination it was also determined whether there were any potential contraindications to exercise.

#### 2.3.2. Physical Performance Measures

Participants’ VO_2_ max values were assessed with the Rockport fitness walking test [30], and the Brzycki equation was applied to predict the 1-RM value of upper- and lower-body maximal strength [31]. Flexibility was measured using a standard bending test executed from the vertical (VB) and the horizontal position (HB) [32].

#### 2.3.3. Self-Report Questionnaire Measures

PAL and SIT were quantified using the IPAQ short-form questionnaire [33], a validated [34] tool that assesses PA level achieved in the previous week, plus other information about time spent in the sitting position (last question of the questionnaire). According to the IPAQ scoring manual [27], IPAQ data were converted into METs, assigning to each activity the conventionally accepted intensity levels: 3.3 METs for walking, 4 METs for moderate-intensity activity, and 8 METs for vigorous-intensity activity. For example, walking energy expenditure (MET-WALK) was derived by multiplying results from walking minutes × walking days × 3.3, while moderate-intensity activity energy expenditure (MET-MOD) was derived by multiplying moderate-intensity activity minutes × number of days of moderate activity × 4.0. Similarly, vigorous-intensity activity energy expenditure (MET-VIG) was derived by multiplying results from vigorous-intensity activity minutes × number of days of vigorous-intensity activity × 8.0. The final score (the energy expenditure related to total physical activity), calculated as MET-WALK + MET-MOD + MET-VIG, is expressed in MET-minutes per week, subsequently transformed into MET-h per week.

### 2.4. Data Analysis

Descriptive analyses in terms of means, standard deviations, and/or percentages were carried out for each variable before (T0) the intervention (all the data are available in Table S1a–c, in Supplementary Materials). The percentage of adherence to exercise intervention was calculated as number of sessions performed/total number of sessions × 100.

The univariate analysis of variance (ANOVA) test was run to compare all variables at baseline, across the BMI, PAL, and SIT categories. To evaluate the effects of the exercise program a repeated-measures multivariate analysis of variance was used. Delta (Δ) changes (T1–T0) were computed and studied through univariate ANOVA of all the measures, using BMI, PAL, and SIT categories as a between factor. Post hoc analysis was conducted, using a Bonferroni correction.

*p*-Values ≤ 0.05 were set as statistically significant. Effect size was measured using partial eta-squares [35]. All the data were digitally archived and the analyses were performed using SPSS^®^ Software, version 25.0 (IBM Corp. Released 2017. IBM SPSS Statistics for Windows, Version 25.0. Armonk, NY, USA: IBM Corp.).

### 2.5. Sample Size Calculation

A sample size of 293 subjects achieves 94% power to detect a mean of paired differences of 15 min on weekly total time of physical activity with an estimated standard deviation of differences of 74 min and with a significance level (alpha) of 0.05 using a two-sided paired *t*-test.

The sample size calculation was performed using PASS Software (PASS 16 Power Analysis and Sample Size Software 2018, NCSS, LLC., Kaysville, UT, USA).

## 3. Results

The percentage of adherence to exercise intervention was 86.9% ± 10.28% for the entire sample. There were no differences between subgroup values.

After the intervention (Table 1), an overall improvement in PAL was observed in the entire sample following the three-month intensive exercise program, with an increase of weekly total time (minutes) dedicated to vigorous physical activity (*p* < 0.001), moderate physical activity (*p* = 0.001), and walking activity (*p* = 0.010). Moreover, a general improvement in several clinical (SBP, *p* < 0.001; DBP, *p* < 0.001; GLYC, *p* < 0.001; TRIG, *p* < 0.001), anthropometric (weight, *p* < 0.001; BMI, *p* < 0.001; WC, *p* < 0.001), and body composition (FM%, *p* < 0.001; MM, *p* = 0.048) variables was observed. Finally, an improvement in physical performance measures (VO_2_ max, *p* < 0.001; body strength, *p* < 0.001; flexibility, *p* < 0.001) occurred.

Please see Table 2a for the results of repeated-measures multivariate analysis of variance to analyze the differences in all variables between T0 and T1, using BMI categories as a between factor.

Using PAL categories as a between factor (Table 2b), the entire sample showed a statistically significant improvement in SBP (*p* < 0.001), DBP (*p* < 0.001), GLYC (*p* = 0.041), TRIG (*p* = 0.001), weight (*p* < 0.001), BMI (*p* < 0.001), WC (*p* < 0.001), FM% (*p* < 0.001), physical performance measures (*p* < 0.001), and weekly energy expenditure related to total physical activity (*p* < 0.001).

The PAL group factor impacted BMI (*p* = 0.047), lat (*p* = 0.038) and chest press (*p* = 0.019), leg extension (*p* = 0.016), weekly energy expenditure related to total physical activity (*p* < 0.001), and daily sitting time (*p* < 0.001).

Using SIT categories as a between factor (Table 2c), the entire sample showed a statistically significant improvement in SBP (*p* < 0.001), DBP (*p* < 0.001), GLYC (*p* < 0.001), COL (*p* = 0.008), TRIG (*p* = 0.001), anthropometric and body composition variables (*p* < 0.001), physical performance measures (*p* < 0.001), weekly energy expenditure related to total physical activity (*p* < 0.001), and daily sitting time (*p* = 0.012). The SIT group factor impacted weight (*p* = 0.013), weekly total physical activity energy expenditure (*p* < 0.001), and daily sitting time (*p* < 0.001).

## 4. Discussion

This study aimed to evaluate the effects of an intensive exercise program of 25 biweekly sessions on the variation of some clinical, anthropometric, body composition, physical performance, and self-report questionnaire variables in a group of overweight and obese adults with and without DM2. We also studied the role played by BMI, PAL, and SIT on the effects of exercise intervention. As expected, we observed an overall improvement of PAL in the entire samples following the three-month intensive exercise program, with a general improvement in several health-related outcomes reviewed; baseline PAL and SIT did not seem to influence all these effects in a sample composed of overweight and obese participants with and without DM2. Similar to the results of other authors [36,37,38,39,40], in our sample, we observed a significative reduction of some clinical and anthropometric variables linked to cardiovascular risk. In fact, as did Cheng et al. [40] and Schwingshackl et al. [41], we observed a significant weight and fat mass reduction, using mixed exercise. With regard to waist circumference, a central adiposity variable useful for identifying specific cardiometabolic risk [42], we observed a significative reduction in the entire sample (*p* < 0.001), even if the post-intervention mean values (107.45 ± 11.73) remained dangerous for health. As also found by Stoner et al. [43], the effects of exercise on LDL, HDL, and HbA1c were inconclusive (*p* > 0.005). As expected, using BMI categories as a between factor, we observed that the previous parameters were influenced by baseline mean values of BMI. In fact, we observed the greatest improvements in subjects with a greater degree of obesity rather than in the overweight group (Table 2a). Particularly, the most important reduction of WC was observed in participants with II degrees (or superior) of obesity rather than in participants with I degree of obesity (*p* = 0.006). In our study, weight and fat mass loss, as well as WC reduction, seemed not to be influenced by PAL and SIT categories as a between factor, with the only exception being the SIT group factor, which impacted weight (low sitting time group vs. high sitting time group, *p* = 0.007). In fact, deepening the weight trend with respect to the SIT groups, we observed a greater reduction in people with low SIT (−3.42 kg) rather than in people with medium (−3.04 kg) and high (−2.85 kg) SIT. These results could be influenced by the SIT trend observed in different SIT groups, although people with low baseline SIT presented a greater increase (+2.49 h per day) rather than those in the medium (−0.39 h per day) and high (−1.50 h per day) groups.

Previous studies of the overweight and obese involved in exercise programs showed improvements in physical measures. Dieli-Conwright et al. [44] found important improvements in estimated VO_2_ max (52%) and muscular strength (>30%), as did Hsu et al. [45], who recorded increases in maximal exercise capacity and maximal muscular strength. Balducci et al. [46,47] reported positive changes in VO_2_ max, upper- and lower-body strength, and flexibility. In our study, we observed statistical changes in VO_2_ max (p < 0.001), upper- (lat machine and chest press test, *p* < 0.001) and lower-body strength (leg extension and leg press test, *p* < 0.001) and flexibility (in horizontal bending and vertical bending test, *p* < 0.001) in the entire sample. These results are largely expected and obvious, and the improvements are due to the combined workouts that stimulate the systems more than what happens in activities of daily living. Using BMI categories as a between factor, we noted that baseline BMI values influenced VO_2_ max (*p* < 0.001), lower- (*p* < 0.001) and upper-body strength (*p* ≤ 0.001), and HB (*p* = 0.021). In these variables, we observed a lower improvement in people with overweight than in the other two groups, according to previous literature concerning obesity’s impact on muscular strength [48].

In our sample, baseline PAL (Table 2b) categories appeared to influence the effects of exercise on lat (*p* = 0.038) and chest press (*p* = 0.019) and leg extension (*p* = 0.016) test values. In fact, we observed greater improvement in people with higher baseline PAL than in people in the others two groups. We could postulate that it can be linked to the fact that the highest performing people were able to load more kilograms during strength exercises, thanks to greater resistance to effort. The SIT (Table 2c) category factor did not seem to influence these variables. Such results must encourage us to promote exercise interventions in all people, independently of body weight and SIT. It is essential, however, to tailor exercise programs for the obese by paying attention to different effort perception and motivation in obese people vs. in normal weight people [49,50]. For obese people, their physical condition is reported as a barrier to exercise; difficulties related to the physical condition of obesity may reduce the rhythm of daily activities, such as walking or exercise.

Appropriate management of the health of overweight and obese adults [51] with and without DM2 should include physical activities and exercise prescription [52]. To better tailor exercise prescription [53], assessment of PAL and SIT represents an essential first step. Unfortunately, clinical settings seem to be in increasingly short supply due to scarce time and economic resources, and these constraints often necessitate a simple, low-cost, rapid assessment tool. Even though some authors have explained that self-reported data are often subject to biases and poor agreement between objective and subjective measures of physical activity has been reported [12,34], the IPAQ-SF is a validated tool [34], used in many clinical settings such as ours. In our study, we collected participants’ self-reported measures (such as PAL and SIT information) through the IPAQ-SF questionnaire.

High amounts of sedentary time (daily/weekly sitting time) have been associated with a significantly greater risk for metabolic syndrome and DM2 [22,54,55,56,57]. As found by Balducci et al. (2019) [58], who reported that an exercise intervention strategy resulted in increased physical activity level and decreased sedentary time, in our study, we observed an improvement in PAL and a decrease in SIT for the entire sample, although the SIT reduction was not statistically significant.

Limitations. This study has some limitations. First, our work did not include a control group. Further, some outcomes were based on self-reported questionnaire measures. To overcome this problem, at least in part, we carefully selected an internationally validated tool (IPAQ). Moreover, we did not use objective measurements (i.e., accelerometry) during exercise sessions. It is also necessary to underline that individuals with musculoskeletal disorders or other clinical conditions that could contraindicate exercise were excluded from the analyses. This aspect may affect the generalizability of our findings. Additionally, in this study, we did not present nutritional data. In future studies, an analysis of eating habits should be carried out before and after the training sessions, given that the nutritional component is relevant in this type of subject. Another limit to underline is the time from data collection to the submission of the manuscript. In the meantime, physical activity programs and new technologies have evolved newer performance and evaluation programs. This could be conditioning the program itself and the result. Finally, we preferred to report the results of this paper using the categories “physical activity level”, “sedentary activity time”, and “BMI” separately. In real time, these factors could be “mixed” with each other (i.e., we can have a high level of physical activity and a high sedentary time in the same subject or a low level of physical activity and a low sedentary time). This scenario could somehow affect the results.

## 5. Conclusions

Currently there is an urgent global need to better understand the improvements in health outcomes derived from reducing SIT and implementing PAL, especially in overweight and obese people who represent a worldwide pandemic emergency. Our study results showed an improvement of PAL in the participants following the three-month intensive exercise program, as well as an improvement in several health-related outcomes observed. Our data suggest that baseline PAL and SIT do not seem to influence all the effects observed in a sample composed by overweight and obese participants with and without DM2. These results must encourage us even more to promote exercise interventions in all people, independently from body weight and SIT.

Future investigations that include more objective instruments (i.e., accelerometry) and control groups should be conducted to obtain further evidence through experimental and translational research in order to better inform public health policy, particularly in terms of tailored exercise prescription addressed to people with obesity and/or DM2. Implementations of a supervised exercise intervention—as shown in this study—produced positive results in health-related outcomes in a group of overweight and obese adults with and without DM2. In our opinion, the evidence-based methodology assessments (C.U.R.I.A.Mo. clinical model protocol), including standardized tests to assess physical measures and other variables, are strengths of this study.

## Figures and Tables

**Table 1:** Student t-test values: mean values of all parameters in the entire sample. Data are presented as means ± SDs, after (T0) and before (T1) the exercise intervention. Statistical significance was set for *p*-values ≤0.05.

Outcomes	T0	T1	t	*p*
	Mn ± SD	Mn ± SD		
SBP	**134.31 ± 17.32**	125.06 ± 14.95	−5.547	<0.001
DBP	82.71 ± 8.80	75.37 ± 10.74	−7.255	<0.001
GLYC	120.22 ± 43.44	110.41 ± 35.68	−3.444	<0.001
HbA1c	7.55 ± 10.13	6.25 ± 0.98	−1.435	0.077
COL	200.03 ± 39.52	193.73 ± 39.74	−2.598	0.005
HDL	47.83 ± 9.87	46.75 ± 10.12	−1.584	0.058
LDL	121.30 ± 36.43	122.04 ± 34.68	0.264	0.396
TRIG	154.64 ± 92.81	135.02 ± 81.17	−3.529	<0.001
WEIGHT	95.61 ± 17.25	92.73 ± 16.86	−13.478	<0.001
BMI	33.59 ± 4.49	32.58 ± 4.41	−12.518	<0.001
WC	111.71 ± 12.02	107.45 ± 11.73	−13.565	<0.001
FM%	38.20 ± 7.63	36.50 ± 7.84	−9.090	<0.001
MM	56.11 ± 11.97	55.83 ± 11.60	−1.671	0.048
LAT	39.04 ± 11.28	48.52 ± 12.25	22.897	<0.001
CHEST	27.92 ± 9.95	38.65 ± 12.13	29.754	<0.001
PRESS	155.24 ± 37.43	202.05 ± 45.96	20.290	<0.001
LEXT	31.17 ± 11.07	47.31 ± 14.27	26.586	<0.001
VB	−8.72 ± 9.84	−4.79 ± 10.45	10.623	<0.001
HB	25.80 ± 10.54	29.33 ± 9.34	9.563	<0.001
VO_2_ max	19.52 ± 9.30	25.93 ± 7.93	16.409	<0.001
MET-h per week	16.46 ± 24.71	39.80 ± 27.88	12.372	<0.001
VIG_TOT_WEEK_MIN	36.20 ± 126.39	175.54 ± 141.12	14.010	<0.001
VIG_ MET-h per week	4.83 ± 16.85	23.41 ± 18.82	14.010	<0.001
MOD_TOT_WEEK_MIN	73.63 ± 187.45	121.37 ± 208.41	3.071	0.001
MOD_ MET-h per week	4.91 ± 12.50	8.09 ± 13.89	3.071	0.001
WALK_TOT_WEEK_MIN	122.31 ± 175.35	151.01 ± 188.41	2.321	0.010
WALK_ MET-h per week	6.73 ± 9.64	8.31 ± 10.36	2.321	0.010
SIT	5.36 ± 3.17	5.15 ± 2.69	−1.056	0.146

SBP: systolic blood pressure; DBP: diastolic blood pressure; GLYC: fasting plasma glucose; HbA1c: glycosylated hemoglobin; COL: total cholesterol; HDL: high-density lipoprotein; LDL: low-density lipoprotein; TRIG: triglycerides; BMI: body mass index; FM%: fat mass percentage; MM: muscle mass; LAT: lat machine test value; CHEST = chest press test value; PRESS = leg press test value; LEXT = leg extension test value; VB: vertical bending test value; HB: horizontal bending test value; VO_2_ max: maximal oxygen consumption value; MET-h per week = weekly total physical activity energy expenditure; VIG_TOT_WEEK_MIN = weekly total time (in minutes) of vigorous physical activity; VIG_ MET-h per week = weekly vigorous physical activity energy expenditure; MOD_TOT_WEEK_MIN = weekly total time (in minutes) of moderate physical activity; MOD_ MET-h per week = weekly moderate physical activity energy expenditure; WALK_TOT_WEEK_MIN = weekly total time (in minutes) of walking activity; WALK_ MET-h per week = weekly walking activity energy expenditure; SIT = daily sitting time.

**Table 2:** Repeated-measures multivariate analysis of variance values, using BMI (**a**), PAL (**b**) and SIT (**c**).

**a**. Repeated-measures multivariate analysis of variance to analyze differences in all variables between T0 and T1, using BMI categories as a between factor. Data are presented as means ± SDs. Statistical significance was set for *p*-values ≤0.05.									
**Outcomes**	**T0**	**T1**	**Time T0 vs. T1**			Time * BMI Category			**Δ (T1–T0) Post Hoc**
	**Mn ± SD**	**Mn ± SD**	**F**	***p***	**Partial η^2^**	**F**	***p***	**Partial η^2^**	
**SBP**	134.31 ± 17.32	125.06 ± 14.95	31.447	<0.001	0.202	3.252	0.615	0.008	N.S.
**DBP**	82.71 ± 8.80	75.37 ± 10.74	49.821	<0.001	0.287	3.949	0.022	0.060	N.S.
**GLYC**	120.22 ± 43.44	110.41 ± 35.68	13.706	<0.001	0.099	0.160	0.852	0.003	N.S.
**HbA1c**	7.55 ± 10.13	6.25 ± 0.98	2.760	0.099	0.023	1.639	0.199	0.027	N.S.
**COL**	200.03 ± 39.52	193.73 ± 39.74	6.666	0.011	0.049	1.588	0.208	0.024	N.S.
**HDL**	47.83 ± 9.87	46.75 ± 10.12	2.809	0.96	0.022	1.238	0.294	0.019	N.S.
**LDL**	121.30 ± 36.43	122.04 ± 34.68	0.029	0.864	0.000	1.069	0.347	0.018	N.S.
**TRIG**	154.64 ± 92.82	135.02 ± 81.17	11.871	0.001	0.083	2.285	0.106	0.034	N.S.
**WEIGHT**	95.61 ± 17.25	92.73 ± 16.86	158.465	<0.001	0.353	123.116	<0.001	0.459	II OB vs. OVER (*p* < 0.001); II OB vs. I OB (*p* < 0.001); I OB vs. OVER (*p* < 0.001)
**BMI**	33.60 ± 4.49	32.58 ± 4.41	135.345	<0.001	0.318	429.297	<0.001	0.748	II OB vs. OVER (p < 0.001); II OB vs. I OB (*p* < 0.001); I OB vs. OVER (*p* < 0.001)
**WC**	111.71 ± 12.02	107.45 ± 11.73	174.209	<0.001	0.389	127.413	<0.001	0.482	II OB vs. OVER (*p* < 0.001); II OB vs. I OB (*p* < 0.001); I OB vs. OVER (*p* < 0.001)
**FM%**	38.20 ± 7.63	36.50 ± 7.84	72.460	<0.001	0.201	36.690	<0.001	0.203	II OB vs. OVER (*p* < 0.001); II OB vs. I OB (*p* < 0.001); I OB vs. OVER (*p* < 0.001)
**MM**	56.11 ± 11.97	55.83 ± 11.60	1.545	0.215	0.005	17.443	<0.001	0.108	II OB vs. OVER (*p* < 0.001); II OB vs. I OB (*p* < 0.001); I OB vs. OVER (*p* < 0.046)
**LAT**	39.04 ± 11.28	48.53 ± 12.25	453.070	<0.001	0.673	6.993	0.001	0.060	II OB vs. OVER (*p* < 0.001)
**CHEST**	27.92 ± 9.95	38.65 ± 12.14	769.477	<0.001	0.778	7.308	<0.001	0.063	II OB vs. OVER (*p* < 0.001); II OB vs. I OB (*p* = 0.044)
**PRESS**	155.24 ± 37.43	202.05 ± 45.96	360.794	<0.001	0.621	12.104	<0.001	0.099	II OB vs. OVER (*p* < 0.001); II OB vs. I OB (*p* < 0.001)
**LEXT**	31.17 ± 11.07	47.31 ± 14.27	621.215	<0.001	0.743	9.548	<0.001	0.082	II OB vs. OVER (*p* < 0.001); II OB vs. I OB (*p* = 0.004)
**VB**	−8.72 ± 9.84	−4.79 ± 10.45	109.377	<0.001	0.279	1.822	0.164	0.013	N.S.
**HB**	25.80 ± 10.54	29.33 ± 9.34	82.575	<0.001	0.226	3.920	0.021	0.027	II OB vs. I OB (*p* = 0.019)
**VO_2_ max**	19.52 ± 9.30	25.93 ± 7.93	231.054	<0.001	0.443	14.542	<0.001	0.091	II OB vs. OVER (*p* < 0.001); II OB vs. I OB (*p* < 0.001)
**MET-h per week**	16.46 ± 24.71	39.80 ± 27.88	136.159	<0.001	0.320	1.113	0.330	0.008	N.S.
**SIT**	5.36 ± 3.17	5.15 ± 2.69	0.714	0.399	0.003	1.704	0.184	0.012	N.S.
**b.** Repeated-measures multivariate analysis of variance to analyze differences in all variables between T0 and T1, using PAL categories as a between factor. Data are presented as means ± SDs. Statistical significance was set for *p*-values ≤0.05.									
**Outcomes**	**T0**	**T1**	**Time T0 vs. T1**			Time * PAL Category			**Δ (T1–T0) Post Hoc**
	**Mn ± SD**	**Mn ± SD**	**F**	***p***	**Partial η^2^**	**F**	***p***	**Partial η^2^**	
**SBP**	134.31 ± 17.32	125.06 ± 14.95	18.319	<0.001	0.129	.395	0.674	0.006	N.S.
**DBP**	82.71 ± 8.80	75.37 ± 10.74	35.135	<0.001	0.221	2.847	0.062	0.044	N.S.
**GLYC**	120.22 ± 43.44	110.41 ± 35.68	4.264	0.041	0.536	1.170	0.314	0.018	high vs. low (*p* = 0.041)
**HbA1c**	7.55 ± 10.13	6.25 ± 0.98	0.972	0.326	0.008	0.599	0.551	0.010	N.S.
**COL**	200.03 ± 39.52	193.73 ± 39.74	3.272	0.073	0.025	2.132	0.123	0.032	N.S.
**HDL**	47.83 ± 9.87	46.75 ± 10.12	0.190	0.663	0.002	0.227	0.797	0.004	N.S.
**LDL**	121.30 ± 36.43	122.04 ± 34.68	0.058	0.811	0.001	1.270	0.285	0.021	N.S.
**TRIG**	154.64 ± 92.82	135.02 ± 81.17	10.978	0.001	0.078	0.019	0.981	0.001	N.S.
**WEIGHT**	95.61 ± 17.25	92.73 ± 16.86	127.497	<0.001	0.305	0.269	0.765	0.002	N.S.
**BMI**	33.60 ± 4.49	32.58 ± 4.41	107.147	<0.001	0.270	3.100	0.047	0.021	moderate vs. low (*p* = 0.046)
**WC**	111.71 ± 12.02	107.45 ± 11.73	112.938	<0.001	0.292	0.582	0.560	0.004	N.S.
**FM%**	38.20 ± 7.63	36.50 ± 7.84	63.138	<0.001	0.180	1.120	0.328	0.008	N.S.
**MM**	56.11 ± 11.97	55.83 ± 11.60	0.374	0.542	0.001	1.141	0.321	0.008	N.S.
**LAT**	39.04 ± 11.28	48.53 ± 12.25	375.487	<0.001	0.631	3.330	0.038	0.029	high vs. moderate (*p* = 0.033)
**CHEST**	27.92 ± 9.95	38.65 ± 12.14	719.275	<0.001	0.767	4.059	0.019	0.036	high vs. moderate (*p* = 0.017); high vs. low (*p* = 0.037)
**PRESS**	155.24 ± 37.43	202.04 ± 45.96	334.875	<0.001	0.604	2.492	0.085	0.022	high vs. moderate (*p* = 0.014); high vs. low (*p* = 0.027)
**LEXT**	31.171 ± 11.07	47.31 ± 14.275	568.074	<0.001	0.725	4.199	0.016	0.038	high vs. moderate (*p* = 0.017); high vs. low (*p* = 0.022)
**VB**	−8.72 ± 9.84	−4.79 ± 10.45	73.522	<0.001	0.206	3.166	0.830	0.001	N.S.
**HB**	25.80 ± 10.54	29.33 ± 9.34	45.052	<0.001	0.137	2.274	0.105	0.016	N.S.
**VO_2_ max**	19.52 ± 9.30	25.93 ± 7.93	158.397	<0.001	0.353	2.474	0.086	0.017	N.S.
**MET-h per week**	16.46 ± 24.71	39.80 ± 27.88	35.445	<0.001	0.109	96.579	<0.001	0.400	high vs. moderate (*p* < 0.001); high vs. low (*p* < 0.001); moderate vs. low (*p* < 0.001)
**SIT**	5.36 ± 3.17	5.15 ± 2.69	0.597	0.440	0.002	8.522	<0.001	0.057	high vs. low (*p* < 0.001); moderate vs. low (*p* = 0.017)
**c**. Repeated-measures multivariate analysis of variance to analyze differences in all variables between T0 and T1, using SIT categories as a between factor. Data are presented as means ± SDs. Statistical significance was set for *p* values ≤ 0.05.									
**Outcomes**	**T0**	**T1**	**Time T0 vs. T1**			Time * SIT Category			**Δ (T1–T0) Post Hoc**
	**Mn ± SD**	**Mn ± SD**	**F**	***p***	**Partial η^2^**	**F**	***p***	**Partial η^2^**	
**SBP**	134.52 ± 17.44	125.31 ± 15.11	21.800	<0.001	0.157	0.055	0.983	0.001	N.S.
**DBP**	83.1 ± 8.83	75.76 ± 10.71	45.168	<0.001	0.279	0.717	0.544	0.018	N.S.
**GLYC**	121.75 ± 43.87	111.46 ± 36.03	19.283	<0.001	0.140	0.281	0.839	0.007	N.S.
**HbA1c**	7.62 ± 10.38	6.26 ± 0.99	1.364	0.245	0.012	0.752	0.524	0.020	N.S.
**COL**	200.37 ± 38.86	193.88 ± 39.62	7.204	0.008	0.055	0.520	0.669	0.013	N.S.
**HDL**	47.80 ± 9.98	46.67 ± 10.28	3.451	0.066	0.028	0.850	0.469	0.021	N.S.
**LDL**	122.248 ± 36.55	122.719 ± 34.40	0.002	0.962	0.024	1.072	0.364	0.029	N.S.
**TRIG**	154.87 ± 94.23	136.13 ± 82.52	11.146	0.001	0.083	0.770	0.513	0.018	N.S.
**WEIGHT**	95.793 ± 17.28	92.92 ± 16.92	148.702	<0.001	0.344	3.678	0.013	0.038	low vs. high SIT (*p* = 0.007)
**BMI**	33.62 ± 4.50	32.61 ± 4.42	125.817	<0.001	0.308	2.244	0.083	0.023	N.S.
**WC**	111.78 ± 12.09	107.56 ± 11.78	153.989	<0.001	0.366	1.936	0.124	0.021	N.S.
**FM%**	38.17 ± 7.59	36.46 ± 7.82	67.703	<0.001	0.194	1.223	0.302	0.013	N.S.
**MM**	56.24 ± 11.95	55.96 ± 11.59	3.245	0.073	0.011	2.505	0.059	0.026	N.S.
**LAT**	39.19 ± 11.33	48.65 ± 12.27	430.401	<0.001	0.668	0.735	0.532	0.010	N.S.
**CHEST**	27.95 ± 9.96	38.64 ± 12.08	752.288	<0.001	0.779	1.322	0.268	0.018	N.S.
**PRESS**	155.59 ± 37.29	202.34 ± 45.99	340.782	<0.001	0.614	2.106	0.100	0.029	N.S.
**LEXT**	31.33 ± 11.07	47.45 ± 14.37	572.982	<0.001	0.733	0.799	0.496	0.011	N.S.
**VB**	−8.66 ± 9.87	−4.71 ± 10.50	108.815	<0.001	0.283	1.061	0.366	0.011	N.S.
**HB**	25.9 ± 10.62	29.44 ± 9.38	83.961	<0.001	0.233	0.417	0.741	0.005	N.S.
**VO_2_ max**	19.69 ± 9.24	25.95 ± 7.96	223.868	<0.001	0.442	1.304	0.273	0.014	N.S.
**MET-h per week**	16.61 ± 24.88	39.95 ± 27.97	134.693	<0.001	0.322	6.841	<0.001	0.068	low SIT vs. very high SIT (*p* = 0.004); low SIT vs. high SIT (*p* < 0.001); medium SIT vs. high SIT (*p* = 0.026)
**SIT**	5.36 ± 3.17	5.15 ± 2.69	6.374	0.012	0.022	271.280	<0.001	0.742	low SIT vs. very high SIT (*p* < 0.001); low SIT vs. high SIT (*p* < 0.001); low SIT vs. medium SIT (*p* < 0.001); medium SIT vs. very high SIT (*p* < 0.001); medium SIT vs. high SIT (*p* < 0.001); high SIT vs. very high SIT vs. (*p* < 0.001); very high SIT vs. high SIT (*p* < 0.001)

SBP: systolic blood pressure; DBP: diastolic blood pressure; GLYC: fasting plasma glucose; HbA1c: glycosylated hemoglobin; COL: total cholesterol; HDL: high-density lipoprotein; LDL: low-density lipoprotein; TRIG: triglycerides; BMI: body mass index; FM%: fat mass percentage; MM: muscle mass; LAT: lat machine test value; CHEST = chest press test value; PRESS = leg press test value; LEXT = leg extension test value; VB: vertical bending test value; HB: horizontal bending test value; VO_2_ max: maximal oxygen consumption value; MET-h per week = weekly total physical activity energy expenditure; SIT = daily sitting time. Between-group comparisons are reported in the last column of the table. Statistically significant differences are then followed by post hoc results (e.g., OVER vs. I OB means that Δ in people with overweight is different from that in the group of people with I degree of obesity). N.S. = not statistically significant. Between-group comparisons are reported in the last column of table. Statistically significant differences are then followed by post hoc results (e.g., high vs. moderate means that Δ in people with high level of physical activity is different from that in people with moderate level of physical activity). N.S. = not statistically significant.

## Data Availability

The datasets used and/or analyzed during the current study are available from the corresponding author on reasonable request.

## Supplementary Materials

The following are available online at https://www.mdpi.com/article/10.3390/jfmk7010012/s1, Table S1a–c. Baseline values: mean values of all parameters in the entire sample and in the subgroups.
