# Supplementary material for: Impact of BMI, Physical Activity, and Sitting Time Levels on Health-Related Outcomes in a Group of Overweight and Obese Adults with and without Type 2 Diabetes
Source: J Funct Morphol Kinesiol. 2022 Jan 17;7(1):12. doi: 10.3390/jfmk7010012 (PMC8788475; doi:10.3390/jfmk7010012)
Supplement: Supplementary file 1 [file jfmk-07-00012-s001.zip › jfmk-1543447-supplementary .pdf]

Table S1a. Baseline values: mean values of all parameters in the entire sample and in the BMI subgroups. Data are presented as means ± SDs. Statistical significance was set for p values ≤ 0.05.

**Outcomes**	**Total Sample** n = 293	**OVER** n = 63	**I OB** n = 131	**II OB** n = 99	**F**	***p***
Age	53.08±9.07	57.43±7.89	57.76±8.75	50.73±9.29	11.411	**<.001**
SBP	135.05±17.43	133.68±11.10	132.52±22.7	139.04±13.26	1.907	.153
DBP	82.75±8.63	80.44±6.2	81.57±8.89	85.85±9.13	4.960	**.008**
GLYC	119.95±43.08	124.52±42.65	114.98±38.33	122.64±48.90	0.626	.536
HbA1c	6.61±1.35	6.86±1.38	6.44±1.25	6.61±1.42	0.926	.399
COL	200.19±39.25	191.74±45.22	200.77±42.56	205.84±28.37	1.267	.285
HDL	47.74±10.05	48.85±8.89	48.58±10.94	45.96±9.74	1.092	.339
LDL	121.71±36.14	117.51±40.16	121.69±81.03	125.12±28.52	0.402	.670
TRIG	153.98±92.29	127.38±63.04	146.60±81.03	182.46±114.63	3.943	**.022**
WEIGHT	95.61±17.25	79±8.96	92.08±11.84	110.84±14.87	135.576	**<.001**
BMI	33.59±4.49	28.04±1.31	32.46±1.48	38.63±2.99	528,954	**<.001**
WC	111.61±12.02	100.02±7.46	108.92±7.32	122.39±10.52	141.425	**<.001**
FM%	38.19±7.62	32.76±6.8	37.57±6.68	42.42±6.87	39.806	**<.001**
MM	56.08±11.96	50.56±9.34	54.94±11.12	61.03±12.67	17.455	**<.001**
LAT	39.11±10.92	35.56±9.21	38.84±10.76	41.70±11.54	6.298	**.002**
CHEST	27.98±9.67	25.23±8.44	27.19±9.07	30.73±10.53	7.153	**<.001**
PRESS	157.54±36.09	148.67±34.73	154.57±35.81	167.01±35.56	5.850	**.003**
LEXT	31.02±10.72	28.61±9.05	29.16±9.80	34.88±11.79	10.438	**<.001**
VB	-8.7±9.83	-9.33±10.16	-7.69±9.75	-9.66±9.68	1.284	.278
HB	25.83±10.54	26.46±10.86	27.18±9.86	23.59±10.95	3.399	**.035**
VO_2 max	19.52±9.3	23.39±9.44	20.48±8.82	15.79±8.57	15.534	**<.001**
Adherence	86.9±10.28	88.2±9.19	86.4±10.38	86.8±10.82	.611	**.544**
MET-h per week	16.46±24.71	15.32±16.92	18.26±26.80	14.81±26.03	0.634	.531
SIT	5.36±3.17	5.34±2.99	5.22±3.22	5.55±3.23	0.296	.744

OVER= people with overweight; I OB= people with I degree of obesity; IIOB= people with II degrees (or superior) of obesity.
SBP: systolic blood pressure; DBP: diastolic blood pressure; GLYC: fasting plasma glucose; HbA1c: glycosylated hemoglobin; COL: total cholesterol; HDL: high-density lipoprotein; LDL: low-density lipoprotein; TRIG: triglycerides; BMI: body mass index; FM%: fat mass percentage; MM: muscle mass; LAT: Lat Machine test value; CHEST = Chest press test value; PRESS = leg press test value; LEXT = leg extension test value; VB: vertical bending test value; HB: horizontal bending test value; VO_2 max: maximal oxygen consumption value; Adherence = the percentage of adherence to exercise intervention (calculated as number of sessions performed / total number of sessions); MET-h per week = weekly total physical activity energy expenditure; SIT = daily sitting time.

Table S1b. Baseline values: mean values of all parameters in the entire sample and in the PAL subgroups. Data are presented as means ± SDs. Statistical significance was set for p values ≤ 0.05.

Outcomes	Total Sample *n* = 293	high PAL *n* = 32	moderate PAL *n* = 108	low PAL *n* = 153	F	*p*
**Age**	53.08±9.07	50.72±9.56	54.30±9.08	52.71±8.89	2.199	.113
**SBP**	135.05±17.43	132.67±17.10	135.93±21.97	134.86±12.69	0.211	.810
**DBP**	82.75±8.63	79.33±8.21	83.49±8.61	82.92±8.69	1.399	.250
**GLYC**	119.95±43.08	137.13±47.86	117.07±43.54	118.31±41.23	1.366	.259
**HbA1c**	6.61±1.35	7.06±1.27	6.48 ±1.38	6.62±1.34	1.041	.356
**COL**	200.19±39.25	184.73 ± 19.86	207.55 ± 39.78	197.36± 41.16	2.363	.098
**HDL**	47.74±10.05	46.43 ± 9.20	46.91 ± 8.73	48.76 ± 11.28	0.619	.540
**LDL**	121.71±36.14	109.55 ± 18.18	127.07 ± 35.82	119.85 ± 38.88	1.372	.258
**TRIG**	153.98±92.29	157.80 ± 133.18	153.79 ± 95.80	153.25 ± 78.48	0.015	.985
**WEIGHT**	95.61±17.25	98.93 ± 16.54	93.84 ± 17.10	96.16 ± 17.46	1.241	.291
**BMI**	33.59±4.49	34.01± 4.32	32.73±4.31	34.12±4.58	3.244	**.040**
**WC**	111.61±12.02	112.78±11.88	110.41±12.38	112.22±11.8	0.887	.413
**FM%**	38.19±7.62	37.35±7.66	37.64±7.46	38.76±7.73	0.890	.412
**MM**	56.08±11.96	58.69±11.57	55.50±11.83	55.93±12.14	0.901	.407
**LAT**	39.11±10.92	43.15±12.49	38.00±9.93	39.09±11.14	2.635	.073
**CHEST**	27.98±9.67	31.22±12.04	27.06±9.51	27.98±9.18	2.195	.113
**PRESS**	157.54±36.09	165.69±33.93	154.38±34.78	158.17±37.33	1.200	.303
**LEXT**	31.02±10.72	35.06±11.70	30.67±9.56	30.50±11.21	2.242	.108
**VB**	-8.7±9.83	-6.34±9.76	-7.57±9.64	-9.99±9.84	2.976	.053
**HB**	25.83±10.54	29.25±11.65	26.52±10.12	24.62±10.45	2.948	.054
**VO_2 max**	19.52±9.3	21.57±8.92	20.82±9.48	18.18±9.11	3.484	**.032**
**Adherence**	86.9±10.28	86.3±10.13	88±10.09	86.1±11.65	.935	.394
**MET-h per week**	16.46±24.71	71.35±32.69	20.35±11.81	2.24±2.84	379.543	**<.001**
**SIT**	5.36±3.17	4.06±2.35	5.10±2.8	5.80±3.48	4.508	**.012**

SBP: systolic blood pressure; DBP: diastolic blood pressure; GLYC: fasting plasma glucose; HbA1c: glycosylated hemoglobin; COL: total cholesterol; HDL: high-density lipoprotein; LDL: low-density lipoprotein; TRIG: triglycerides; BMI: body mass index; FM%: fat mass percentage; MM: muscle mass; LAT: Lat Machine test value; CHEST = Chest press test value; PRESS = leg press test value; LEXT = leg extension test value; VB: vertical bending test value; HB: horizontal bending test value; VO2 max: maximal oxygen consumption value; Adherence = the percentage of adherence to exercise intervention (calculated as number of sessions performed / total number of sessions); MET-h per week = weekly total physical activity energy expenditure; SIT = daily sitting time.

Table S1c. Baseline values: mean values of all parameters in the entire sample and in the SIT subgroups. Data are presented as means ± SDs. Statistical significance was set for p values ≤ 0.05.

**Outcomes**	**Total Sample** *n* = 293	**very high SIT** *n* = 43	**high SIT** *n* = 99	**medium SIT** *n* = 63	**low SIT** *n* = 82	**F**	***p***
Age	52.84±9.01	52.19±9.54	53.81±8.65	55.08±9.75	50.30±8.02	4.043	**.008**
SBP	135.29±17.54	130.50±29.88	136.95±13.64	133.96±11.82	137.13±15.04	0.859	.464
DBP	83.04±8.64	86.00±8.40	82.20±7.83	80.96±7.62	83.64±9.94	1.575	.199
GLYC	121.43±43.49	131.00±61.96	122.82±40.76	121.20±41.41	114.79±34.74	0.666	.575
HbA1c	6.64±1.38	7.09±1.94	6.75±1.37	6.57±1.30	6.32±0.98	1.467	.227
COL	200.53±39.57	206.41±41.98	194.15±37.31	207.52±45.15	198.95±36.63	0.807	.492
HDL	47.71±10.16	44.95±8.48	47.41±8.78	49.15±12.74	48.36±10.31	0.708	.549
LDL	122.66±36.24	129.55±37.27	115.41±34.93	132.60±40.50	119.89±33.39	1.401	.246
TRIG	154.17±93.67	189.10±118.35	146.54±86.95	149.26±109.51	146.98±71.26	1.172	.323
WEIGHT	95.79±17.28	101.81±20.77	96.22±16.21	91.63±17.25	95.32±15.85	3.066	**.028**
BMI	33.62±4.5	34.53±5.06	33.80±4.16	32.58±4.68	33.72±4.37	1.808	.146
WC	111.69±12.09	114.71±14.70	112.09±11.52	109.90±12.33	111.02±10.95	1.459	.226
FM%	38.17±7.58	37.43±8.51	39.14±7.50	37.86±7.16	37.61±7.50	0.862	.461
MM	56.21±11.94	60.38±13.84	55.54±11.47	53.96±11.38	56.54±56.54	2.653	**.049**
LAT	39.27±10.94	41.35±11.80	39.38±11.47	38.29±8.92	38.78±11.28	0.739	.529
CHEST	28.06±9.63	29.74±9.52	27.16±9.17	26.89±8.62	29.16±10.80	1.372	.252
PRESS	158.01±35.90	173.89±33.10	156.89±35.48	154.92±33.47	153.55±37.89	3.434	**.017**
LEXT	31.17±10.72	33.41±10.47	31.05±11.47	29.77±8.63	31.23±11.30	0.940	.422
VB	-8.64±9.86	-10.98±11.81	-9.44±9.78	-8.16±9.71	-6.80±8.73	2.004	.114
HB	25.94±10.62	22.95±12.21	25.78±10.51	25.98±10.19	27.66±10.00	1.835	.141
VO_2 max	19.69±9.24	20.79±8.39	18.97±9.50	19.51±9.56	20.12±9.18	0.466	.706
Adherence	86.8±10.29	87.3±8.90	88.2±9.55	86±11.04	85.8±11.94	0.780	.506
MET-h per week	16.61±24.88	6.65±9.80	13.60±18.81	22.71±33.94	20.79±26.76	5.015	**.002**
SIT	5.36±3.17	10.44±1.48	6.81±0.83	4.62±0.48	1.49±1.24	815.446	**<.001**

SBP: systolic blood pressure; DBP: diastolic blood pressure; GLYC: fasting plasma glucose; HbA1c: glycosylated hemoglobin; COL: total cholesterol; HDL: high-density lipoprotein; LDL: low-density lipoprotein; TRIG: triglycerides; BMI: body mass index; FM%: fat mass percentage; MM: muscle mass; LAT: Lat Machine test value; CHEST = Chest press test value; PRESS = leg press test value; LEXT = leg extension test value; VB: vertical bending test value; HB: horizontal bending test value; VO2 max: maximal oxygen consumption value; Adherence = the percentage of adherence to exercise intervention (calculated as number of sessions performed / total number of sessions); MET-h per week = weekly total physical activity energy expenditure; SIT = daily sitting time.
